# Retrospective Comparative Study of Oral Versus Subcutaneous Semaglutide in Patients with Type 2 Diabetes Mellitus

**DOI:** 10.3390/ijms27114694

**Published:** 2026-05-22

**Authors:** Barbara Toffoli, Matteo Michieletto, Stella Bernardi, Riccardo Candido

**Affiliations:** 1 Department of Medical Surgical and Health Sciences, University of Trieste, Ospedale di Cattinara, Strada di Fiume 447, 34149 Trieste, Italy; btoffoli@units.it (B.T.); matteo.michieletto@studenti.units.it (M.M.); riccardo.candido@asugi.sanita.fvg.it (R.C.); 2 SS Endocrinologia (Medicina Clinica), Azienda Sanitaria Universitaria Giuliano Isontina (ASUGI), Ospedale di Cattinara, Strada di Fiume 447, 34149 Trieste, Italy; 3 SC Patologie Diabetiche, Azienda Sanitaria Universitaria Giuliano Isontina (ASUGI), Via Sai 7, 34128 Trieste, Italy

**Keywords:** oral semaglutide, subcutaneous semaglutide, GLP-1RA, type 2 diabetes, comparative study, propensity score matching

## Abstract

Semaglutide represents a unique therapeutic option for patients with type 2 diabetes mellitus (T2DM), being the first and currently only glucagon-like peptide-1 receptor agonist (GLP-1RA) available in both subcutaneous and oral formulations. This study aimed to compare the effectiveness of oral versus subcutaneous (sc) semaglutide on metabolic parameters and cardiovascular risk factors in T2DM patients. This is a retrospective real-world study including adult patients with T2DM taking oral or sc semaglutide followed at the ASUGI Diabetes Center. We analyzed data from 434 patients (median age 70 years, diabetes duration 13 years), treated with oral (n = 232) or sc (n = 202) semaglutide. The oral formulation had a higher discontinuation rate. Among these patients, 130 patients in the oral group and 145 in the sc group had an 18-month follow-up. When comparing these groups, patients taking sc semaglutide had a significantly higher baseline BMI. However, multivariate linear regression models suggested that both formulations were comparably effective in reducing HbA1c and BMI, with baseline values being the primary predictors of response. To address BMI imbalances, propensity score matching was performed, identifying 55 matched pairs. Both oral and sc semaglutide reduced HbA1c and BMI and there were no significant differences in the median change in HbA1c and BMI between groups. Interestingly, oral semaglutide was associated with a significantly greater reduction in diastolic blood pressure compared to the sc formulation. Furthermore, concomitant therapy with SGLT2 inhibitors significantly enhanced the reduction in total and LDL cholesterol. Oral and subcutaneous semaglutide show comparable effectiveness in lowering HbA1c and BMI in a real-world setting.

## 1. Introduction

Type 2 diabetes mellitus (T2DM) remains a global health challenge, requiring therapeutic strategies that not only achieve glycemic control but also promote weight loss and reduce diabetic complications [1]. In this setting, glucagon-like peptide-1 receptor agonists (GLP-1RAs) have emerged as a cornerstone of diabetes therapy [2]. Among these, semaglutide represents a unique therapeutic option, being the first and currently only GLP-1RA available in both subcutaneous and oral formulations.

The clinical efficacy of both formulations has been extensively demonstrated in randomized controlled trials (RCTs), such as the SUSTAIN and PIONEER programs [3,4,5], which reported significant reductions in glycated hemoglobin (HbA1c) and body weight as well as reductions in major adverse cardiovascular events (SUSTAIN-6; PIONEER-6) [6,7]. Recent real-world studies have begun to explore how oral semaglutide compares with the subcutaneous formulation in routine clinical practice, suggesting that the oral formulation is often preferred in older patients or in those transitioning from other oral glucose-lowering drugs, whereas the injectable form is more frequently initiated in younger patients with higher body mass index (BMI) [8,9]. However, direct real-world comparisons between the oral and subcutaneous formulations remain limited, particularly regarding their effects on cardiovascular risk factors beyond glycemic control, such as blood pressure and lipid profiles.

Our group has previously demonstrated the effectiveness of oral semaglutide in diverse clinical scenarios—whether used as an add-on therapy or as a switch from other treatments—showing significant improvements in metabolic parameters [10]. Furthermore, we recently explored the biological determinants of this response [11], evaluating whether specific genetic variants of the GLP-1 receptor (GLP1R) may influence the individual therapeutic response.

Based on this background, the aim of the present study was to compare the clinical effectiveness of oral versus subcutaneous semaglutide in a large, real-world cohort of T2DM patients, looking at metabolic outcomes as well as cardiovascular risk factors, including blood pressure and lipid profiles, in order to provide more data for personalizing GLP-1RA therapy.

## 2. Results

### 2.1. Population Characteristics

A total of 434 patients with T2DM were enrolled, and their baseline characteristics are summarized in Table 1. The study cohort had a median age of 70 years (IQR 63–76) and was predominantly male (56.9%, 247/434). Patients were mostly obese, with a median BMI of 30.9 kg/m^2^ (IQR 27.4–35.5). The median baseline HbA1c was 7.4% (IQR 6.7–8.1), and the median diabetes duration was 13 years (IQR 7–22).

When comparing the treatment groups, 232 patients received oral semaglutide (oral GLP-1RA group) and 202 subcutaneous (sc) semaglutide (scGLP-1RA group). Oral semaglutide was initiated at a dose of 3 mg for the first 4 weeks and subsequently increased, according to glycemic control and gastrointestinal tolerability, to a final dose of 7 mg in 90/232 patients (38.8%) and 14 mg in 117/232 patients (50.4%). For subcutaneous semaglutide, 166/202 patients (32.7%) received the 0.5 mg dose, while 136/202 (67.3%) were treated with the 1 mg dose. As reported in Table 1, the treatment groups differed significantly at baseline with respect to BMI and diabetes duration. Only 32/434 patients (7.4%) were prescribed semaglutide as monotherapy. In most cases, both oral and subcutaneous semaglutide were prescribed in replacement of another glucose-lowering drug: 157/232 patients (67.7%) in the oral GLP-1RA group and 135/202 patients (66.8%) in the scGLP-1RA group. Notably, oral semaglutide was more frequently combined with SGLT2 inhibitors, whereas subcutaneous semaglutide was more commonly used alongside metformin and sulphonylureas.

Treatment was discontinued in 60/232 patients (25.9%) in the oral GLP-1RA group and in 13/202 patients (6.4%) in the scGLP-1RA group (*p* < 0.001). The most common reasons for discontinuation were gastrointestinal side effects, mainly vomiting and diarrhea. However, other reported adverse events included diaphoresis, pruritus, and skin rashes. Patients treated with subcutaneous semaglutide occasionally reported minor injection site discomfort or reactions, which were not observed in the oral group. This is consistent with the literature and it may reflect the daily administration schedule of the oral formulation and the higher sensitivity to gastrointestinal tolerability in older patients [12]. Conti et al. have recently demonstrated that medication persistence was significantly lower when semaglutide was administered as a once-daily tablet compared with a weekly injection [9].

### 2.2. 18-Month Effects of Oral vs. Subcutaneous Semaglutide on Metabolic Parameters and Cardiovascular Risk Factors

In the initial cohort, 130 patients in the oral GLP-1RA group and 145 patients in the scGLP-1RA group had an 18-month follow-up. Table 2 summarizes their baseline characteristics.

**Figure 1:**
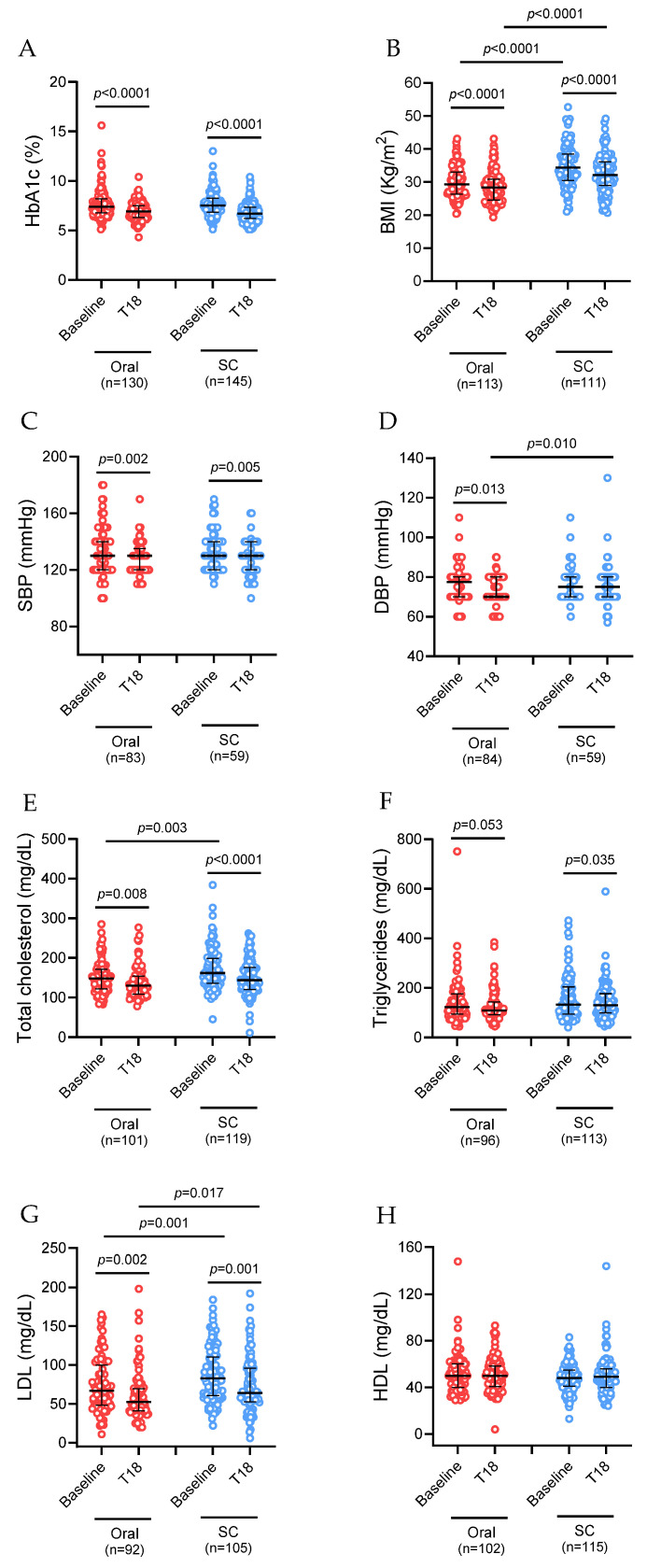
Effects of oral and subcutaneous semaglutide after 18 months of follow-up. (**A**) HbA1c, glycated hemoglobin; (**B**) BMI, body mass index; (**C**) SBP, systolic blood pressure; (**D**) DBP, diastolic blood pressure; (**E**) total cholesterol; (**F**) triglycerides; (**G**) LDL, low-density lipoprotein cholesterol; (**H**) HDL, high-density lipoprotein cholesterol. Oral, oral semaglutide; SC, subcutaneous semaglutide.

As shown in Figure 1A, after 18 months of treatment, both oral semaglutide and subcutaneous semaglutide significantly reduced HbA1c from baseline, with median changes of −0.5% (IQR −1.20; 0.10) in the oral GLP-1RA group and −0.7% (IQR −1.30; 0.00) in the scGLP-1RA group. Both treatments also significantly reduced BMI; however, the subcutaneous formulation demonstrated a more pronounced effect. Specifically, the median BMI change was −1.3 kg/m^2^ (IQR −2.15; −0.35) in the oral group compared with −2.0 kg/m^2^ (IQR −3.80; −0.60) in the scGLP-1RA group (*p* = 0.005) (Figure 1B).

Regarding blood pressure (Figure 1C,D), both treatment modalities effectively lowered systolic blood pressure (SBP), whereas a significant reduction in diastolic blood pressure (DBP) was observed only in the oral GLP-1RA group. Furthermore, treatment with either oral or subcutaneous semaglutide significantly improved lipid profiles, leading to favorable changes in total cholesterol, triglycerides, and LDL cholesterol (Figure 1E–H).

### 2.3. Impact of the Route of Semaglutide Administration on Metabolic Parameters and Cardiovascular Risk Factors: Multivariate Analysis

A multivariate linear regression analysis was performed to assess whether the route of semaglutide administration influenced changes in HbA1c, BMI, blood pressure, and lipid profile, while accounting for potential confounders. As reported in Table 3, higher baseline BMI and HbA1c were independently associated with greater reductions in HbA1c, whereas concomitant therapy with SGLT2 inhibitors was associated with smaller changes (β = 0.244, *p*= 0.043). Regarding BMI, female patients and those with higher baseline BMI exhibited greater weight loss over the 18-month follow-up period (Table 3).

Both systolic and diastolic blood pressure changes were primarily driven by their respective baseline values (SBP: β = −0.737, *p* < 0.0001; DBP: β = −0.798, *p* < 0.0001). Notably, the oral formulation of semaglutide was independently associated with a greater decrease in DBP compared to the subcutaneous formulation (Table 4). Finally, changes in lipid levels were mainly determined by their baseline values (total cholesterol: β = −0.729, *p* < 0.0001; triglycerides: β = −0.830, *p* < 0.0001; LDL cholesterol: β = −0.749, *p* < 0.0001). Male sex was associated with a greater reduction in total cholesterol (β = −10.244, *p* = 0.045) but a smaller reduction in triglycerides (β = 18.279, *p* = 0.042). Concomitant SGLT2 inhibitor therapy was associated with enhanced reductions in total and LDL cholesterol (β = −15.490, *p* = 0.004; β = −12.849, *p* = 0.009, respectively). Baseline HbA1c independently predicted total cholesterol reduction (β = 4.214, *p* = 0.010), whereas age independently predicted LDL cholesterol reduction (β = −0.525, *p* = 0.021) (Table 4).

### 2.4. Oral vs. Subcutaneous Semaglutide on Metabolic Parameters After Propensity Score Matching

After propensity score matching analysis, we selected 55 patients treated with oral semaglutide and 55 patients treated with subcutaneous semaglutide who did not differ in terms of age, sex, baseline HbA1c and BMI, or diabetes duration (Table 5).

**Figure 2:**
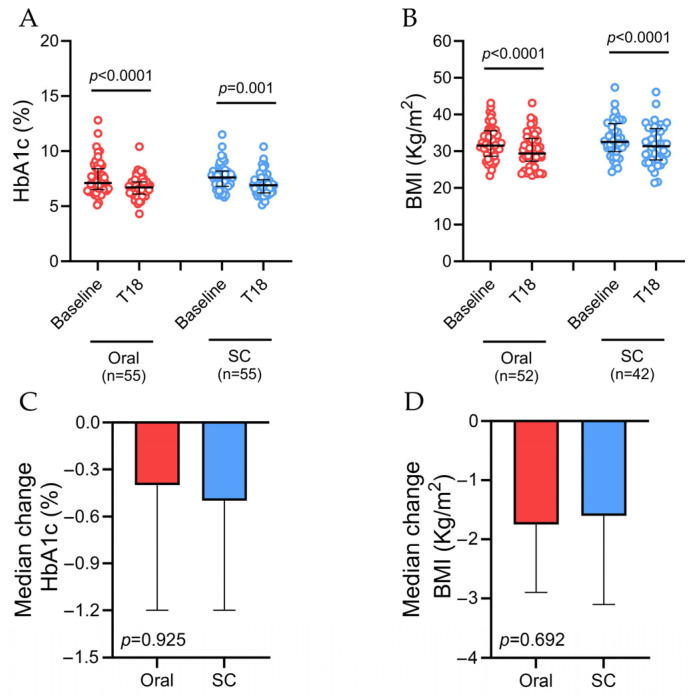
Effects of oral and subcutaneous semaglutide after 18 months of follow-up in patients selected with propensity score matching. Evolution of (**A**) glycated hemoglobin (HbA1c) and (**B**) body mass index (BMI), from baseline to 18 months of follow-up in patients treated with oral and subcutaneous (SC) semaglutide. Median change in HbA1c (**C**) and BMI (**D**) from baseline to 18 months of follow-up in patients treated with oral and subcutaneous (SC) semaglutide.

As shown in Figure 2, after 18 months of treatment, both oral and subcutaneous semaglutide significantly reduced HbA1c from baseline, with median changes of −0.4% (IQR −1.2; 0.1) in the oral GLP-1RA group and −0.5% (IQR −1.2; 0.15) in the scGLP-1RA group. Both treatments also significantly reduced BMI; specifically, the median BMI change was −1.75 kg/m^2^ (IQR −2.8; −0.7) in the oral GLP-1RA group compared with −1.60 kg/m^2^ (IQR −3.1; −0.1) in the scGLP-1RA group. When comparing the changes in HbA1c and BMI between the two groups, no statistically significant differences were observed, indicating a comparable long-term effectiveness for both oral and subcutaneous semaglutide.

A multivariate linear regression analysis was performed to assess whether the route of semaglutide administration influenced changes in HbA1c and BMI, while accounting for potential confounders (Table 6).

As reported in Table 6, patients with higher baseline HbA1c exhibited greater HbA1c reductions (β = −0.714, *p* < 0.0001), whereas concomitant therapy with SGLT2 inhibitors or sulfonylureas was associated with smaller decreases (β = 0.523, *p* < 0.004; β = 0.540, *p* < 0.016, respectively). Regarding BMI, neither the route of administration nor the association with SGLT2 inhibitors had a significant effect on BMI changes over the 18-month follow-up period (Table 6).

## 3. Discussion

The present real-world study provides a comparison between oral and subcutaneous semaglutide in a large cohort of patients with type 2 diabetes (T2DM) followed in a specialized clinical setting. On the one hand, our findings confirm the comparable clinical effectiveness of both formulations in improving glycemic control and metabolic parameters after 18 months of treatment, while showing that oral semaglutide was discontinued more often, mainly due to gastrointestinal side effects. Conversely, our analysis of cardiovascular risk factors suggests that oral semaglutide might be associated with a greater reduction in diastolic blood pressure compared to the subcutaneous formulation. Furthermore, concomitant therapy with SGLT2 inhibitors seems to enhance the reduction in total and LDL cholesterol.

Interestingly, our cohort was significantly older (median age 70 years) than those typically enrolled in randomized controlled trials like PIONEER or SUSTAIN, and even older than recent real-world cohorts from the UK [13]. This suggests that in Italian clinical practice, semaglutide is perceived as a valuable tool for managing older, often polymedicated patients. In addition, when looking at the general characteristics of our patients, a primary finding of our study is the distinct clinical profile that seems to drive the prescription of the two formulations. For instance, we found that subcutaneous semaglutide was preferentially prescribed to obese patients (median BMI 33.4 kg/m^2^) while oral semaglutide was preferentially prescribed to overweight patients (median BMI 29.1 kg/m^2^). This is in line with the observations of Formichi and Conti [8,9], who reported that subcutaneous semaglutide was preferred in patients with higher excess weight and shorter disease duration, while the oral formulation was used later and especially after therapeutic failure of previous therapies. It has been argued that the preference for subcutaneous semaglutide in patients with higher classes of obesity may reflect the weight-reducing efficacy demonstrated in earlier clinical trials [8].

Nevertheless, multivariate analysis showed that both formulations achieved significant BMI reductions, which seemed to be influenced only by baseline BMI rather than the specific semaglutide formulation. This was confirmed by comparing the two groups after PSM analysis. Although the sample was reduced from 434 to 110 patients, PSM allowed us to balance the groups for BMI and diabetes duration. Notably, we found that BMI change was −1.75 kg/m^2^ in the oral GLP-1RA group and −1.60 kg/m^2^ in the scGLP-1RA group. While some studies suggest a potential long-term advantage for the injectable form in weight loss [14], our data, as well as those of other authors [8,9,13], support the concept that both formulations have comparable effectiveness in body weight reduction when adherence is maintained.

Moreover, both oral and subcutaneous semaglutide achieved significant HbA1c reductions, demonstrating that semaglutide remains highly effective even in the later stages of the natural history of diabetes, given that our population had a median age of 70 years and more than 10 years of diabetes duration. Interestingly, our multivariate analysis showed no statistically significant difference in HbA1c changes between oral and subcutaneous semaglutide (*p* = 0.118), once adjusted for baseline characteristics. This was confirmed by comparing the two groups after PSM analysis. In particular, we found that HbA1c change was −0.4% in the oral GLP-1RA and −0.5% in the scGLP-1RA groups. When looking at the SUSTAIN and PIONEER clinical trials, in which HbA1c was lowered by 1–1.4% with oral semaglutide and by 1.2–1.8% with sc semaglutide [15,16], it can be argued that the HbA1c change that we found was smaller. However, the strongest predictor of HbA1c change is its baseline level, which was 7.4% in our study, confirming that the magnitude of GLP-1RA effect is largely dependent on the initial degree of metabolic derangement rather than on the route of administration, as previously reported in our earlier studies [10,11]. In addition, baseline BMI and the use of SGLT2i were also independently associated with HbA1c reduction. Notably, a large proportion of the cohort (34.8%) was prescribed SGLT2 inhibitors, and only 7.4% of patients were prescribed semaglutide as a monotherapy.

As for the traditional cardiovascular risk factors such as blood pressure and lipid profile, the novel result of our study is the association between oral semaglutide and a significantly greater reduction in diastolic blood pressure (DBP) compared to the subcutaneous formulation. While the cardiovascular benefits of GLP-1RAs, particularly on systolic blood pressure, are well-documented [17], specific data on DBP differences between formulations are scarce. Our findings regarding DBP are consistent with the established cardiovascular profile of oral semaglutide. Following the initial cardiovascular safety data from the PIONEER-6 trial [7], the SOUL trial has recently demonstrated a significant 14% reduction in MACE [18], highlighting the cardiovascular benefits of the oral formulation. However, it is important to note that these trials were not designed to compare hemodynamic effects between different semaglutide formulations, and our observational data should be considered hypothesis-generating. In addition, our findings warrant further investigation, as we did not systematically check the concomitant antihypertensive therapy nor its changes during follow-up.

With respect to the lipid profile, our data revealed sex-specific differences in lipid metabolism, with males showing a greater reduction in total cholesterol but a smaller impact on triglycerides compared to females. This aligns with recent evidence from Piccione et al. [19], which emphasized how sex can modulate the response to GLP-1RAs. The dimorphism in body fat distribution and hormonal milieu likely plays a role in how semaglutide reshapes the lipidome, suggesting that a “one-size-fits-all” approach may overlook subtle but important clinical nuances. Finally, our data suggest the presence of a synergy between semaglutide and SGLT2 inhibitors. Concomitant SGLT2 inhibitor therapy was associated with an enhanced reduction in total and LDL cholesterol. However, this association should be interpreted cautiously, as we did not systematically check for concomitant lipid-lowering therapies nor treatment changes during follow-up. Therefore, our findings should be considered hypothesis-generating rather than evidence of a causal synergistic metabolic effect.

The main limitations of this study include its retrospective design and the potential for selection bias inherent to real-world clinical practice (only 7.4% of patients were prescribed semaglutide as a monotherapy). In addition, the baseline clinical characteristics of patients treated with oral versus subcutaneous semaglutide were initially different, particularly regarding BMI and diabetes duration. Although multivariate models were initially used to adjust for these differences, residual confounding could not be entirely excluded. To address this, we implemented a propensity score matching (PSM) analysis, which allowed for a more rigorous comparison between groups. Despite the resulting reduction in sample size, this approach has contributed to improve the internal validity of our comparative effectiveness data. On the other hand, a major strength of our study is the extended 18-month follow-up period, which exceeds the 6-to-12-month duration of most existing real-world studies, providing valuable insights into the long-term durability of the treatment response. Furthermore, the inclusion of cardiovascular risk factors beyond glycemic control—such as blood pressure and lipid profiles—adds new findings to the current literature on semaglutide formulations.

In conclusion, oral and subcutaneous semaglutide show comparable effectiveness in lowering HbA1c and BMI in a real-world setting. Interestingly, oral semaglutide might offer an additional benefit in reducing DBP, while the combination with SGLT2i appears to optimize the lipid profile. These findings highlight the broader benefits of GLP-1RAs including the amelioration of cardiovascular risk factors. On the other hand, the higher discontinuation rate of the oral formulation is a clinical factor that needs to be taken into account as it might influence its overall effectiveness in daily clinical practice.

## 4. Materials and Methods

### 4.1. Study Design

This observational retrospective cohort study aimed to compare the effects of oral semaglutide (oral GLP-1RA) and subcutaneous semaglutide (scGLP-1RA) on metabolic parameters and cardiovascular risk factors in patients with T2DM. It was conducted in accordance with the Declaration of Helsinki, and the protocol was approved by the Institutional Review Board of the University of Trieste, Trieste, Italy (University of Trieste Comitato Etico di Ateneo #136 date 30 November 2023). Its design follows the recommendations of the Strengthening the Reporting of Observational Studies in Epidemiology (STROBE) Statement [20].

Adult patients with T2DM taking oral or subcutaneous semaglutide were screened for eligibility through the electronic medical records of the ASUGI Diabetes Center (S.C. Patologie Diabetiche, ASUGI, Trieste, Italy). Exclusion criteria were: (i) patients under the age of 18; (ii) absence of follow-up data; (iii) non-adherence to prescribed medication; (iv) diagnosis of type 1 diabetes mellitus or gestational diabetes; and (v) lack of informed consent to participate in the study. Once eligible patients were identified, between October 2024 and April 2025 they were asked to sign the consent form during their regularly scheduled outpatient visits. Patients were fully informed about the purpose of the project and provided their written informed consent for the use of their data and inclusion in the study.

In all these patients, we collected baseline and follow-up data on demographic characteristics, diabetes duration, previous and concomitant medications, dose of oral or subcutaneous semaglutide, as well as clinical parameters, which included HbA1c, body mass index (BMI), systolic and diastolic blood pressure (SBP, DBP) and lipid profile. Drug discontinuation and adverse events were also recorded.

**Figure 3:**
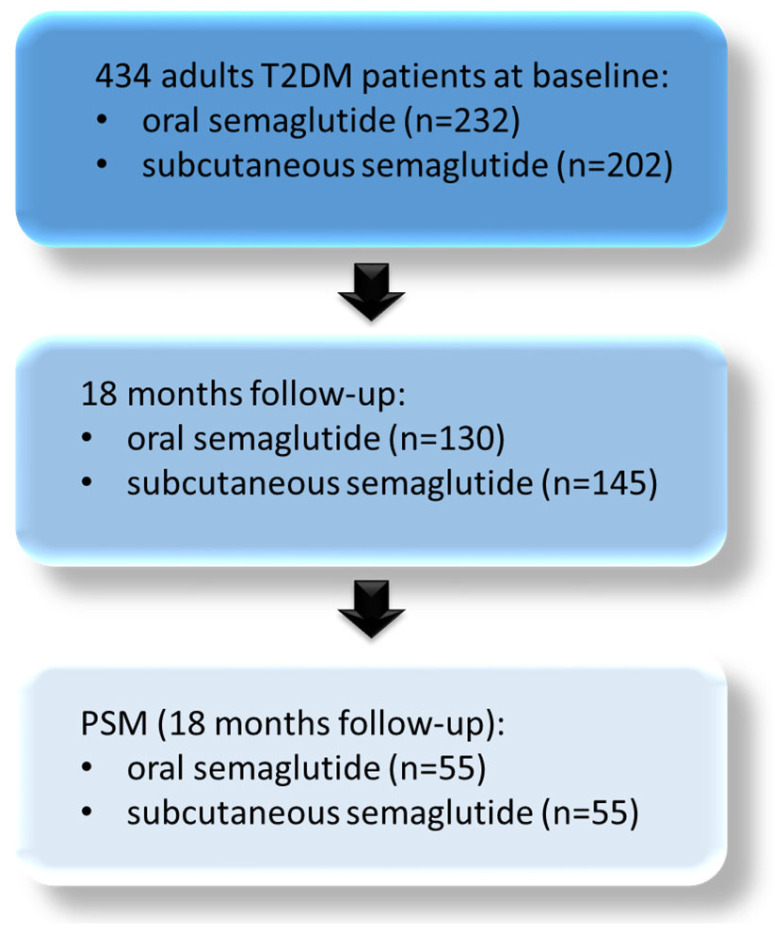
Patient selection flow-chart. PSM, propensity score matching; T2DM, type 2 diabetes mellitus.

Then, for this study, among the 434 patients who provided informed consent, we specifically selected those who had completed an 18-month follow-up period for longitudinal analysis. Subsequently, to ensure a balanced comparison between the two treatment formulations, we performed a propensity score matching (PSM) analysis, which resulted in the selection of 55 pairs of patients (55 in the oral GLP-1RA group and 55 in the scGLP-1RA group), Figure 3.

### 4.2. Statistical Analysis

All statistical analyses were performed using R statistical software (version 4.4.0; R Development Core Team, The R Foundation for Statistical Computing, Vienna, Austria). Statistical significance was set at *p* < 0.05. Continuous variables were reported as median and interquartile range (IQR), and the Shapiro–Wilk test was used to assess normality. Categorical variables were presented as absolute frequencies and percentages. Comparisons of continuous variables were carried out using the *t*-test or the Wilcoxon rank-sum test, depending on data distribution. Paired analyses (baseline vs. 18-month follow-up) were performed using the paired *t*-test or the Wilcoxon signed-rank test. When more than two groups were compared, a two-way ANOVA followed by Šídák’s multiple comparisons test was used. Categorical variables were compared using the chi-square test or Fisher’s exact test, as appropriate.

Associations between drug choice and treatment response were evaluated using linear regression models to adjust for potential confounding variables. The dependent variables in these models were the reductions in HbA1c, BMI, SBP, DBP, total cholesterol, triglycerides, and LDL cholesterol. Models were adjusted for age, sex, baseline HbA1c, baseline BMI, medication type (oral GLP-1RA vs. subcutaneous GLP-1RA), and SGLT2i co-treatment. Baseline SBP, DBP, total cholesterol, triglycerides, and LDL cholesterol were also included when the corresponding variable reduction (SBP, DBP, total cholesterol, triglycerides, or LDL cholesterol) was the dependent variable. Co-treatment with sulfonylureas was included only in the multivariate model for HbA1c reduction.

To compare the patients treated with oral GLP1RA to those treated scGLP-1RA, in order to control for potential confounders and selection bias, we performed a sensitivity analysis using propensity score matching with the R package ‘MatchIt’ version 4.5 (method nearest neighbor). The patients were matched 1:1 by age, sex, HbA1c, BMI and diabetes duration. Linear regression models to adjust for potential confounding variables were performed on this subgroup of patients as previously described.

## Figures and Tables

**Table 1:** Population characteristics.

Variable		All Patients (n = 434)	Oral GLP-1RA (n = 232)	scGLP-1RA (n = 202)	*p*-Value
**Age (years)**		70 (63–76)	71 (63.7–77.2)	69 (62–75)	0.07
**Sex (n; %)**	M	247/434 (56.9%)	132/232 (56.9%)	115/202 (56.9%)	0.99
	F	187/434 (43.1%)	100/232 (43.1%)	87/202 (43.1%)	
**Diabetes duration (years)**		13 (7–22)	12 (6–21)	15 (8–22)	0.028
**Baseline HbA1c (%)**		7.4 (6.7–8.1)	7.3 (6.6–8.1)	7.5 (6.8–8.1)	0.253
**Baseline BMI (kg/m^2^)**		30.9 (27.4–35.5)	29.1 (26.3–33.6)	33.4 (29.5–37.6)	<0.0001
**Dose of oral semaglutide (mg)**	3 mg	25/232 (10.8%)	25/232 (10.8%)	NA	
	7 mg	90/232 (38.8%)	90/232 (38.8%)		
	14 mg	117/232 (50.4%)	117/232 (50.4%)		
**Dose of sc semaglutide (mg)**	0.5 mg	166/202 (32.7%)	NA	166/202 (32.7%)	
	1 mg	136/202 (67.3%)		136/202 (67.3%)	
**Monotherapy (Yes) (n; %)**		32/434 (7.4%)	20/232 (8.6%)	12/202 (5.9%)	0.286
**Semaglutide (n; %)**	SWITCH	292/434 (67.3%)	157/232 (67.7%)	135/202 (66.8%)	0.852
	ADD-ON	142/434 (32.7%)	75/232 (32.3%)	67/202 (33.2%)	
**Metformin (Yes) (n; %)**		306/434 (70.5%)	148/232 (63.8%)	158/202 (78.2%)	0.001
**SGLT2i (Yes) (n; %)**		151/434 (34.8%)	118/232 (50.9%)	33/202 (16.3%)	<0.0001
**Insulin (Yes) (n; %)**		143/434 (32.9%)	78/232 (33.6%)	65/202 (32.2%)	0.750
**SU (Yes) (n; %)**		70/434 (16.1%)	24/232 (10.3%)	46/202 (22.8%)	0.001
**Pioglitazone (Yes) (n; %)**		35/434 (8.1%)	16/232 (6.9%)	19/202 (9.4%)	0.338
**Treatment discontinuation (Yes) (n; %)**		73/434 (16.8%)	60/232 (25.9%)	13/202 (6.4%)	<0.0001
**Reasons for discontinuation (n, %)**	GI adverse events	21/73 (28.8%)	18/60 (30.0%)	3/13 (23.1%)	0.009
	Other adverse events	24/73 (32.9%)	20/60 (33.3%)	4/13 (30.8%)	
	Lack of efficacy	4/73 (5.5%)	4/60 (6.7%)	0/13 (0.0%)	
	Treatment change	11/73 (15.1%)	7/60 (11.7%)	4/13 (30.7%)	
	Injection site reactions	2/73 (2.7%)	0/60 (0.0%)	2/13 (15.4%)	
	ND	11/73 (15.1%)	11/60 (18.3%)	0/13 (0.0%)	

The *p*-value refers to the comparison between the oral GLP-1RA and scGLP-1RA groups. Abbreviations: BMI, body mass index; GI, gastrointestinal; GLP-1RA, glucagon-like peptide-1 receptor agonist; HbA1c, glycated hemoglobin; NA, not applicable; ND, not determined; sc, subcutaneous; SGLT2i, sodium-glucose cotransporter-2 inhibitors; SU, sulphonylureas.

**Table 2:** Baseline characteristics of the patients with 18 months of follow-up.

Variable		Oral GLP-1RA (n = 130)	scGLP-1RA (n = 145)	*p*-Value
**Age (years)**		71 (65–77)	69 (62–75)	0.131
**Sex (n; %)**	M	79/130 (60.8%)	81/145 (55.9%)	0.41
	F	51/130 (39.2%)	64/145 (44.1%)	
**Diabetes duration (years)**		12 (7–21)	15 (9–23)	0.013
**Baseline HbA1c (%)**		7.4 (6.8–8.2)	7.5 (6.9–8.2)	0.633
**Baseline BMI (kg/m^2^)**		29.1 (26.2–33.0)	33.6 (29.8–37.9)	<0.0001
**Dose of oral semaglutide (mg)**	3 mg	5/130 (3.9%)	NA	
	7 mg	34/130 (26.1%)		
	14 mg	91/130 (70.0%)		
**Dose of subcutaneous semaglutide (mg)**	0.5 mg	NA	37/145 (25.5%)	
	1 mg		108/145 (74.5%)	
**Monotherapy (Yes) (n; %)**		8/130 (6.2%)	7/145 (4.8%)	0.629
**Semaglutide (n; %)**	SWITCH	91/130 (70.0%)	100/145 (69.0%)	0.852
	ADD-ON	39/130 (30.0%)	45/145 (31.0%)	
**Metformin (Yes) (n; %)**		84/130 (64.6%)	110/145 (75.9%)	0.041
**SGLT2i (Yes) (n; %)**		72/130 (55.4%)	27/145 (18.6%)	<0.0001
**Insulin (Yes) (n; %)**		43/130 (33.1%)	51/145 (35.2%)	0.714
**SU (Yes) (n; %)**		18/130 (13.8%)	39/145 (26.9%)	0.008
**Pioglitazone (Yes) (n; %)**		10/130 (7.7%)	13/145 (9.0%)	0.703
**Treatment discontinuation (Yes) (n; %)**		12/130 (9.2%)	8/145 (5.5%)	0.236

The *p*-value refers to the comparison between the oral GLP-1RA and scGLP-1RA groups. Abbreviations: BMI, body mass index; GLP-1RA, glucagon-like peptide-1 receptor agonist; HbA1c, glycated hemoglobin; NA, not applicable; sc, subcutaneous; SGLT2i, sodium-glucose cotransporter-2 inhibitors; SU, sulphonylureas.

**Table 3:** Multivariate linear regression analysis on metabolic parameters.

Predictive Variable	HbA1c Change		
	β-estimate	95% CI	*p*-value
**Age**	0.010	[−0.002;0.021]	0.091
**Sex (Male)**	−0.142	[−0.358;0.074]	0.197
**scGLP-1RA (vs. oral GLP-1RA)**	0.105	[−0.134; 0.344]	0.388
**Baseline BMI**	−0.029	[−0.048; −0.010]	0.004
**Baseline HbA1c**	−0.741	[−0.818; −0.664]	<0.0001
**SGLT2i (yes vs. no)**	0.244	[0.008; 0.480]	0.043
**Sulphonylureas (yes vs. no)**	0.208	[−0.067; 0.483]	0.138
**Predictive Variable**	**BMI Change**		
	β-estimate	95% CI	*p*-value
**Age**	0.006	[−0.026; 0.038]	0.724
**Sex (Male)**	0.722	[0.108; 1.336]	0.021
**scGLP-1RA (vs. oral GLP-1RA)**	−0.440	[−1.116; 0.235]	0.200
**Baseline BMI**	−0.134	[−0.191; −0.077]	<0.0001
**Baseline HbA1c**	−0.040	[−0.253; 0.174]	0.714
**SGLT2i (yes vs. no)**	−0.561	[−1.219; 0.096]	0.094

Abbreviations: BMI, body mass index; CI, confidence interval; GLP-1RA, glucagon-like peptide-1 receptor agonist; HbA1c, glycated hemoglobin; sc, subcutaneous; SGLT2i, sodium-glucose cotransporter-2 inhibitors.

**Table 4:** Multivariate linear regression analysis on cardiovascular parameters.

Predictive Variable	SBP Change		
	β-estimate	95% CI	*p*-value
**Age**	0.135	[−0.068; 0.337]	0.191
**Sex (Male)**	−1.291	[−5.289; 2.707]	0.524
**scGLP-1RA (vs. oral GLP-1RA)**	0.142	[−4.363; 4.648]	0.950
**Baseline BMI**	−0.115	[−0.499; 0.269]	0.554
**Baseline HbA1c**	−0.458	[−1.878; 0.962]	0.524
**Baseline SBP**	−0.737	[−0.860; −0.614]	<0.0001
**SGLT2i (yes vs. no)**	−2.192	[−6.469; 2.085]	0.313
**Predictive Variable**	**DBP Change**		
	β-estimate	95% CI	*p*-value
**Age**	−0.070	[−0.229; 0.089]	0.384
**Sex (Male)**	−0.769	[−3.851; 2.312]	0.622
**scGLP-1RA (vs. oral GLP-1RA)**	3.637	[0.153; 7.121]	0.041
**Baseline BMI**	0.025	[−0.269; 0.319]	0.867
**Baseline HbA1c**	−0.747	[−1.844; 0.350]	0.180
**Baseline DBP**	−0.798	[−0.962; −0.633]	<0.0001
**SGLT2i (yes vs. no)**	2.249	[−1.070; 5.567]	0.182
**Predictive Variable**	**Total Cholesterol Change**		
	β-estimate	95% CI	*p*-value
**Age**	−0.117	[−0.628; 0.393]	0.651
**Sex (Male)**	−10.244	[−20.258; −0.230]	0.045
**scGLP-1RA (vs. oral GLP-1RA)**	−2.527	[−13.206; 8.152]	0.641
**Baseline BMI**	0.190	[−0.705; 1.085]	0.676
**Baseline HbA1c**	4.214	[1.005; 7.423]	0.010
**Baseline total cholesterol**	−0.729	[−0.835; −0.623]	<0.0001
**SGLT2i (yes vs. no)**	−15.490	[−25.955; −5.025]	0.004
**Predictive Variable**	**Triglyceride Change**		
	β-estimate	95% CI	*p*-value
**Age**	0.170	[−0.719; 1.059]	0.706
**Sex (Male)**	18.279	[0.702; 35.856]	0.042
**scGLP-1RA (vs. oral GLP-1RA)**	7.052	[−11.704; 25.807]	0.459
**Baseline BMI**	1.128	[−0.451; 2.707]	0.160
**Baseline HbA1c**	3.393	[−2.412; 9.197]	0.250
**Baseline triglycerides**	−0.830	[−0.858; −0.801]	<0.0001
**SGLT2i (yes vs. no)**	−5.815	[−24.390; 12.760]	0.538
**Predictive Variable**	**LDL Cholesterol Change**		
	β-estimate	95% CI	*p*-value
**Age**	−0.525	[−0.968; −0.081]	0.021
**Sex (Male)**	−1.661	[−10.596; 7.275]	0.714
**scGLP-1RA (vs. oral GLP-1RA)**	0.946	[−8.423; 10.315]	0.842
**Baseline BMI**	−0.042	[−0.824; 0.740]	0.916
**Baseline HbA1c**	2.800	[−0.508; 6.109]	0.097
**Baseline LDL cholesterol**	−0.749	[−0.882; −0.616]	<0.0001
**SGLT2i (yes vs. no)**	−12.849	[−22.462; −3.236]	0.009

Abbreviations: BMI, body mass index; CI, confidence interval; GLP-1RA, glucagon-like peptide-1 receptor agonist; HbA1c, glycated hemoglobin; LDL, low-density lipoprotein; sc, subcutaneous; SGLT2i, sodium-glucose cotransporter-2 inhibitors.

**Table 5:** Baseline characteristics of the patients with 18 months of follow-up selected with PSM.

Variable		Oral GLP-1RA (n = 55)	scGLP-1RA (n = 55)	*p*-Value
**Age (years)**		69 (64–77)	71 (67–76)	0.36
**Sex (n; %)**	M	35/55 (63.6%)	38/55 (69.1%)	0.545
	F	20/55 (36.4%)	17/55 (30.9%)	
**Diabetes duration (years)**		14 (8–24)	15 (9–25)	0.51
**Baseline HbA1c (%)**		7.1 (6.5–8.4)	7.6 (6.8–8.1)	0.272
**Baseline BMI (kg/m^2^)**		31.4 (28.4–35.3)	32 (28.8–35.5)	0.763
**Dose of oral semaglutide (mg)**	3 mg	3/55 (5.5%)	NA	
	7 mg	12/55 (21.8%)		
	14 mg	40/55 (72.7%)		
**Dose of subcutaneous semaglutide (mg)**	0.5 mg	NA	13/55 (23.6%)	
	1 mg		42/55 (76.4%)	
**Monotherapy (Yes) (n; %)**		5/55 (9.1%)	4/55 (7.3%)	0.728
**Semaglutide (n; %)**	SWITCH	40/55 (72.7%)	40/55 (72.7%)	1
	ADD-ON	15/55 (23.7%)	15/55 (27.3%)	
**Metformin (Yes) (n; %)**		33/55 (60.0%)	37/55 (67.3%)	0.428
**SGLT2i (Yes) (n; %)**		30/55 (54.5%)	13/55 (23.6%)	0.001
**Insulin (Yes) (n; %)**		21/55 (38.2%)	19/55 (34.5%)	0.692
**SU (Yes) (n; %)**		5/55 (9.1%)	16/55 (29.1%)	0.008
**Pioglitazone (Yes) (n; %)**		3/55 (5.5%)	5/55 (9.1%)	0.463
**Treatment discontinuation (Yes) (n; %)**		6/55 (10.9%)	3/55 (5.5%)	0.297

The *p*-value refers to the comparison between the oral GLP-1RA and scGLP-1RA groups. Abbreviations: BMI, body mass index; GLP-1RA, glucagon-like peptide-1 receptor agonist; HbA1c, glycated hemoglobin; NA, not applicable; sc, subcutaneous; SGLT2i, sodium-glucose cotransporter-2 inhibitors; SU, sulphonylureas.

**Table 6:** Multivariate linear regression analysis in patients selected with propensity score matching.

Predictive Variable	HbA1c Change		
	β-estimate	95% CI	*p*-value
**Age**	0.006	[−0.012;0.025]	0.504
**Sex (Male)**	−0.261	[−0.622;0.099]	0.153
**scGLP-1RA (vs. oral GLP-1RA)**	0.284	[−0.073; 0.641]	0.118
**Baseline HbA1c**	−0.714	[−0.843; −0.585]	<0.0001
**SGLT2i (yes)**	0.523	[0.165; 0.880]	0.004
**Sulphonylureas (yes)**	0.540	[0.100; 0.980]	0.016
**Predictive Variable**	**BMI Change**		
	β-estimate	95% CI	*p*-value
**Age**	0.001	[−0.044; 0.048]	0.943
**Sex (Male)**	0.489	[−0.363; 1.342]	0.257
**scGLP-1RA (vs. oral GLP-1RA)**	0.163	[−0.690; 1.016]	0.704
**Baseline BMI**	−0.062	[−0.149; −0.025]	0.160
**SGLT2i (yes vs. no)**	−0.762	[−1.621; 0.096]	0.081

Abbreviations: BMI, body mass index; CI, confidence interval; GLP-1RA, glucagon-like peptide-1 receptor agonist; HbA1c, glycated hemoglobin; sc, subcutaneous; SGLT2i, sodium-glucose cotransporter-2 inhibitors.

## Data Availability

The original contributions presented in this study are included in the article. Further inquiries can be directed to the corresponding author.
